# Controlling the Optical and Electrical Properties of Perovskite Films and Enhancing Solar Cell Performance Using the Photonic Curing Process

**DOI:** 10.3390/nano14231975

**Published:** 2024-12-09

**Authors:** Moulay Ahmed Slimani, Arjun Wadhwa, Luis Felipe Gerlein, Jaime A. Benavides-Guerrero, Mohamad Hassan Taherian, Ricardo Izquierdo, Sylvain G. Cloutier

**Affiliations:** 1 Département de Génie Électrique, École de Technologie Supérieure, 1100 Rue Notre-Dame Ouest, Montréal, QC H3C 1K3, Canada; moulay-ahmed.slimani.1@ens.etsmtl.ca (M.A.S.); arjun.wadhwa.1@ens.etsmtl.ca (A.W.); luis-felipe.gerlein-reyes@lacime.etsmtl.ca (L.F.G.); jaime-alberto.benavides-guerrero.1@ens.etsmtl.ca (J.A.B.-G.); sylvaing.cloutier@etsmtl.ca (S.G.C.); 2 Département de Génie Mécanique, Université du Québec à Trois-Rivières, 3351 Boul. des Forges, Trois-Rivières, QC G9A 5H7, Canada; mohamad.hassan.taherian@uqtr.ca

**Keywords:** perovskite solar cell, photonic curing, transient photocurrent, photoluminescence

## Abstract

The most common method of processing metal oxide and perovskite thin films in the laboratory is thermal annealing (TA), which is a constraint for the commercialization of large-scale perovskite solar cells. Here, we present a photonic curing (PC) process to produce fully photonically annealed perovskite cells—a fast process with well-controlled, short light pulses—to develop perovskite photovoltaic devices with high efficiency. We also demonstrate how to use the parameters of the photonic annealing system to control the optical, electrical, morphological, and structural properties of perovskite layers for photovoltaic device applications. The effect of PC treatment on the microstructure, granularity, and electronic properties was studied by scanning electron microscopy (SEM), photoluminescence (PL), and transient photocurrent (TPC). The degree of conversion of the perovskite precursor and its influence on the electronic structure have been identified. $SnO_{2}$ and perovskite films were treated with a single pulse and produced PCE comparable to control samples treated by TA.

## 1. Introduction

Perovskite solar cells (PSCs) have shown remarkable results in terms of power conversion efficiency (PCE), which has exceeded 26.7% in the last decade [1]. This success is mainly due to the perovskite material, which is considered a promising candidate for thin-film solar cells due to its low-cost processing capability [2,3] and excellent optoelectronic properties. These properties include superior light absorption over a wide range of wavelengths in the solar spectrum, due to their tunable bandgap and long charge carrier (electrons, holes) diffusion length [4,5,6]. These characteristics allow perovskite cells to achieve energy conversion efficiencies comparable to, or even surpassing, those of other established solar cells, such as silicon and cadmium telluride (CdTe) [2,7]. Currently, these energy conversion records are obtained in laboratories primarily by using TA to convert precursors into a crystalline perovskite structure. However, this technique is incompatible with roll-to-roll (R2R) fabrication and presents a major drawback for large-scale production of perovskite solar cells, limiting their contribution to the transition to renewable energy [8,9]. Additionally, TA, which is based on equilibrium heating, can cause failures in flexible plastic devices (which are sensitive to high temperatures) due to the mismatch of the thermal coefficient of expansion (TCE) between the layers and the substrate. It is therefore essential to replace TA with PC in the PSC manufacturing process, as PC is compatible with R2R manufacturing. Due to their quick processing times and low energy requirements, photo-irradiation techniques such as near-infrared radiation [10,11], flash infrared annealing [12,13], laser annealing [14,15], and ultraviolet light have recently been researched as alternatives to TA in PSC manufacturing. PC has significantly reduced annealing time, from 10 min for TA to a few milliseconds for PC, which could lower associated costs. Recent studies have used PC or intense pulsed light to crystallize perovskite precursors into a crystalline phase in just a few milliseconds [11,16,17]. This technique has not been limited to the perovskite active layer but has also been extended to other PSC layers, such as transparent metal oxides (TMOs) like $TiO_{2}$ [18,19], $SnO_{2}$ [8,20], and NiO [21,22], enabling the fabrication of a complete PSC using PC. The PC delivers short, intense pulses of light from a broadband xenon flash lamp (200–1500 nm). The difference in absorption between the perovskite precursors and the substrate allows for selective heating, with the energy being absorbed primarily by the perovskite film without damaging the substrate.

Previous studies have investigated the potential for printed carbon-based perovskite solar cells to be enhanced in terms of performance through the use of humidity-assisted heat treatment [23]. By precisely controlling relative humidity and temperature conditions during the annealing process, the quality of perovskite films is improved, leading to more efficient and stable devices. The results demonstrate a reduction in defects in the crystalline structure and a notable enhancement in photovoltaic performance, while maintaining a cost-effective, metal-free manufacturing process. Furthermore, it is established that the identification of optimal materials and the precise timing of antisolvent ejection significantly improve the quality of the perovskite film, leading to enhanced PCE and long-term device reliability [24].

In this work, the combined effect of antisolvent treatment and photonic annealing on perovskite conversion is investigated. We used different antisolvents to create an intermediate phase that facilitates crystallization by increasing the heating capacity of the perovskite precursors. The antisolvents used are chlorobenzene (CB), ethyl ether (EE), and a mixture of chlorobenzene and ethyl ether (CB:EE) with a molar ratio of (3:1). The objective of this study is to evaluate the performance of fully photonically annealed perovskite solar cells (PSCs) compared to thermally annealed PSCs. This will provide insight into the impact of PC parameters on the optical, electrical, morphological, and structural properties of perovskite films. By examining these effects, we aim to elucidate and control the properties of perovskite layers. The findings clearly show that optimizing these parameters will lead to the development of more efficient and cost-effective solar cells with significant potential for large-scale commercialization.

## 2. Experimental Section

$PbI_{2}$ was purchased from Alfa, and methylammonium iodide from GreatCell Solar. $SnO_{2}$ was obtained from Alfa Aesar (15% in $H_{2}O$ colloidal dispersion, CN: 044592.A3); the particles were diluted with DI water to a concentration of 3% by volume. Patterned fluorine-doped tin Oxide (FTO) substrates were purchased from sf-international (SHENZHENHUAYU UNION TECHNOLOGY, Shenzhen, China), and all other materials were sourced from Sigma-Aldrich (Oakville, ON, Canada). All purchased products were used as received. FTO-coated glass substrates (15 ohm/sq) were cleaned sequentially in soapy water, acetone, IPA, and DI water for 10 min and dried with $N_{2}$. They were then treated with $O_{2}$ plasma (Plasma Etch, Carson City, NV, USA, PE-100LF) for 15 min. For perovskite ink, a 0.85 M solution was prepared by mixing equimolar amounts of MAI and $PbI_{2}$ in anhydrous DMSO. The hole transport material 2,2,7,7-tetrakis(N,N-dimethoxyphenylamine) 9,9-spirobifluorene (Spiro-OMeTAD) was dissolved in anhydrous chlorobenzene (CB) at a concentration of 80 mg L^−1^ and doped with 25 mL lithium bis(trifluoromethanesulfonyl)imide (LiTFSI) (170 mg L^−1^ in acetonitrile) and 28.8 μL of 4-tert-butylpyridine (tBP) solution. $SnO_{2}$ was spin-coated onto the glass substrate at 3000 rpm for 30 s in an ambient environment. For TA, the films were annealed at 150 °C for 30 min. For PC, the films were cured using the parameters (3.55 J·cm^−2^, 3500 ms) presented in our previous work [25]. For both TA and PC, the perovskite precursor solution (70 mL) was spin-coated on the $SnO_{2}$ (treated with $O_{2}$ plasma) at 4000 rpm for 30 s inside an $N_{2}$-purged glovebox. The antisolvent (150 μL) was applied to the film 15 s before the end of spin-coating (SC). For PC, the films were prepared inside the same glovebox and processed using PulseForge (500 V, 3 A) power supply, three-capacitor bank with a maximum radiant energy delivery of 20 J·cm^−2^, xenon flash lamp with 150 mm × 75 mm illumination area, and a wide bandwidth (200–1500 nm). For TA samples, $MAPbI_{3}$ films were annealed at 100 °C for 10 min in the glovebox. Afterwards, a spiro-OMeTAD-based HTL was spin-coated at 2000 rpm for 30 s. Finally, a 100 nm Ag electrode was thermally evaporated at a pressure of $10^{-6}$ torr to complete the device. The PSC configuration and materials are identical for TA and PC except for the annealing process used for $SnO_{2}$ and perovskite. For PC, the perovskite films were all processed in air at ambient temperature (22–24 °C) and relative humidity (25–40%). UV-Vis absorption, SEM, FTIR, and PL were used for characterization. For PL, the spectra were normalized by reducing the duration to 1 s and laser power to 1 mW. The full width at half maximum (FWHM) and the position of the maximum were obtained by fitting the spectra with a Gaussian function. XRD was conducted using a Bruker D8 Advance (Billerica, MA, USA), equipped with a Cu source. The temperature is simulated using NovaCentrix SimPulse software (simulation package is standard on the PulseForge Version 3, TX, USA). The configuration was modeled from bottom to top, with the following layers: aluminum chuck 6 mm, glass 2.2 mm, FTO 600 nm, and 300 nm of perovskite. The glass and FTO layer thicknesses were supplied by the manufacturer. Current density–voltage (J-V) measurements were carried out under an AM 1.5 G 100 mW ·cm^−2^ solar simulator (Oriel, MA, USA). All other electrical measurements were taken using PAIOS (Fluxim AG, SN:20121 Winterthur, Switzerland).

## 3. Results and Discussion

**Figure 1:**
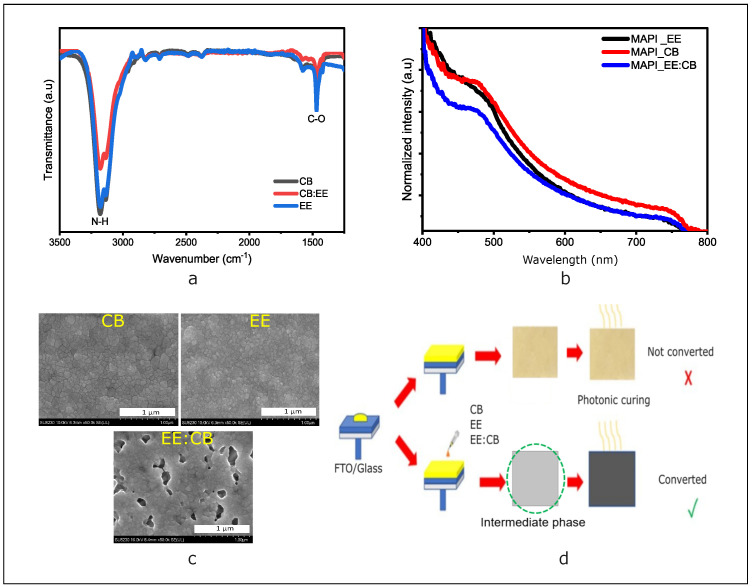
(**a**) FTIR spectra of perovskite films with different solvents, CB (black line), EE (blue line), and CB:EE (red line); (**b**) UV-Vis absorption spectra of MAPI films obtained from different antisolvents CB (red line), EE (black line), and EE:CB (blue line); (**c**) SEM images of surface of MAPI films prepared by CB antisolvent, diethyl-ether antisolvent, and a mix of CB and EE at a ratio 1:1. (**d**) Illustration of PC process.

FTIR, SEM, and absorbance characterizations presented in Figure 1 enabled us to choose the appropriate solvent for our photonic process. FTIR analysis of the solvents studied shows the appearance of the first broad peak at 3180 cm^−1^, corresponding to the stretching of the N-H bond, while the narrow peak is associated with the C-O bond at 1468 cm^−1^ (Figure 1a) [26,27], confirming the MAPI signature for all the solvents studied. The intensity of the FTIR peaks and the absorption spectrum (Figure 1a,b) are both significantly greater for the CB solvent. Figure 1c illustrates that the film treated with this solvent is more compact, uniform, and exhibits larger grains. However, films treated with EE and CB display small grains and surface defects (holes), respectively. To understand how PC affects the electrical and optical properties of perovskite films, the crystallinity and morphology of the films were studied. MAPI films are generally light yellow, which makes the photonic annealing process on glass difficult due to its transparency. First, we studied the effect of solvents on the crystallinity and morphology of the films, finding that the CB solvent creates an intermediate phase, and the color becomes darker, facilitating light absorption and consequently perovskite crystallization, as shown in Figure 1d. The effect of the solvent is not limited to an instant color change (intermediate phase), but also affects granularity and microstructure [28]. The improved quality of the active layer highlights the importance of interface quality in optimizing device performance [29,30].

The optical absorbance of perovskite films is a critical element for evaluating the conversion of precursors into a crystalline structure. We measured the absorbance of perovskite films produced by PC immediately after SC. It is known that the crystallization process is spontaneous in air, as film crystallinity and surface morphology are sensitive to environmental conditions [31,32]. To understand and elucidate how PC parameters affect the crystallinity of MAPI films, we performed photonic annealing of perovskite films at different energy densities and pulse durations, followed by absorbance measurements. This approach enabled us to optimize the PC parameters and map the distinct MAPI conversion zones. We employed an absorbance criterion at a wavelength of 774 nm to compare the absorbance of PC films with that of the reference thermally annealed film [16].

**Figure 2:**
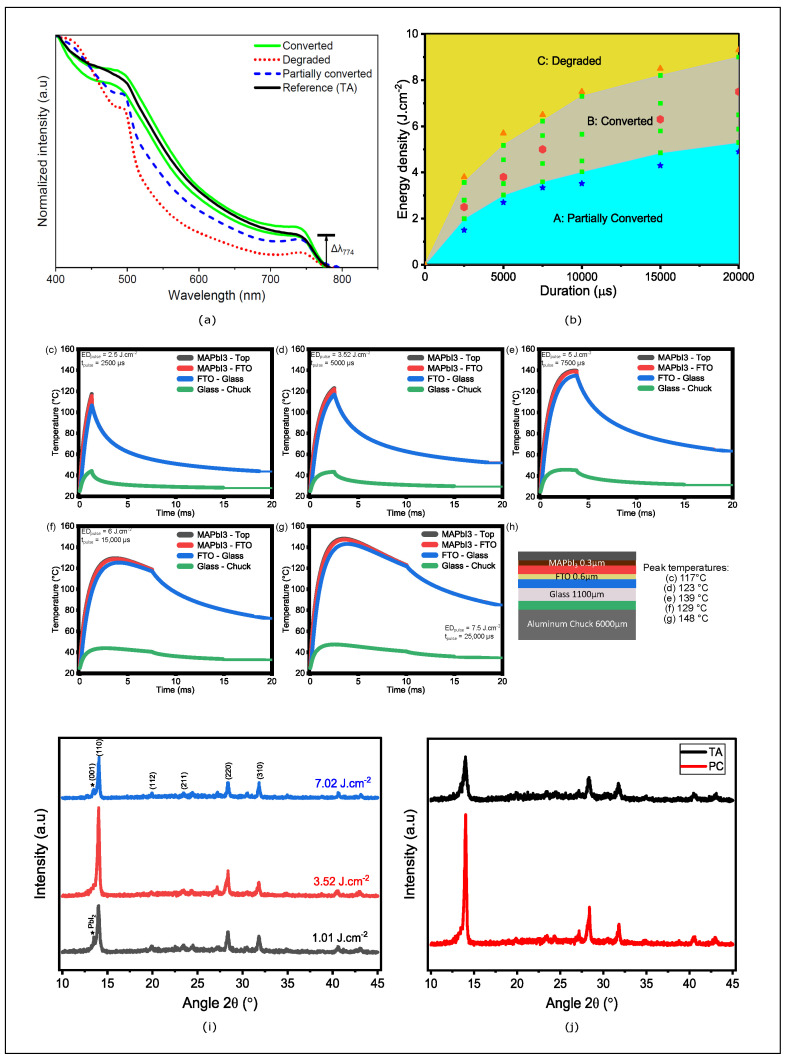
(**a**) Absorbance spectra of TA MAPI films (black line) partially converted PC (dashed blue line) when Δ$A_{774}$ of the PC is below 90% of the TA reference (low energy density), converted PC (green line) when Δ$A_{774}$ of the PC is superior to 90% (medium energy density), and degraded PC (dashed red line) when Δ$A_{774}$ is inferior to 90% (high energy density). (**b**) Mapping of photonically annealed perovskite films based on criterion (**a**), partially converted (turquoise zone), fully converted (gray zone), and degraded (yellow zone). SimPulse simulations of MAPI film temperature profiles at pulse lengths and density energies corresponding to the center of the conversion zone (red hexagon); (**c**) 2.5 J·cm^−2^/2500 μs; (**d**) 3.52 J·cm^−2^/5000 μs; (**e**) 5 J·cm^−2^/7500 μs; (**f**) 6 J·cm^−2^/15,000 μs; (**g**) 7.5 J·cm^−2^/20,000 μs. (**h**) The configuration used for the temperature simulation, and the maximum temperature for each photonic annealing. (**i**) XRD patterns of MAPI films deposited on a glass substrate and photonically annealed at 5000 μs pulse duration and energy densities of 1.01 J·cm^−2^ (black line), 3.52 J·cm^−2^ (red line), and 7.02 J·cm^−2^ (blue line), * corresponds to the diffraction of the $PbI_{2}$ peak associated with the (001) plane. (**j**) XRD patterns for the MAPI films, the thermally annealed reference film (black line), and the photonically annealed film at 5000 μs and 3.52 J·cm^−2^ (red line).

Figure 2 shows the change in absorbance of photonically annealed films. The energy density delivered to the film has a significant impact on the film’s conversion status. When the energy density is low, Δ$A_{774}$ of the PC is below 90% of the TA reference (Figure 2a, blue curve), indicating that the film is partially converted. Conversely, when the energy density is increased, Δ$A_{774}$ exceeds 90% (Figure 2a, green curve), signifying that the film has fully converted. However, at a certain energy density threshold, the perovskite film begins to degrade, exhibiting low absorbance (Figure 2a, red curve). The use of UV-Vis absorbance has enabled the mapping of three different conversion zones of perovskite precursors, which were achieved using PC’s two independent variables: pulse duration and energy density. The results of the temperature simulation, as illustrated in Figure 2c–g, demonstrate a complex relationship between energy density, pulse duration, and the resulting temperature on the perovskite film when photonic annealing parameters are employed. The applied energy density ranges from 2.5 to 7.5 J·cm^−2^, and the pulse duration varies from 2500 to 25,000 μs. These parameters are of great consequence in determining the energy transferred to the perovskite film. In general, an increase in energy density and pulse duration results in an elevation of temperature, although the effect is not entirely linear. The recorded temperatures on the perovskite film ranged from 117 to 148 degrees Celsius (Figure 2h). In the initial stages, an increase in energy density and pulse duration results in a corresponding rise in temperature. For example, at an energy density of 2.5 J·cm^−2^ and a pulse duration of 2500 μs, the temperature is 117 °C. An increase in energy density to 3.52 J·cm^−2^ and pulse duration to 7500 μs results in a temperature rise to 139 °C. However, a non-linear behavior is observed when the energy density reaches 6 J·cm^−2^ with a pulse duration of 15,000 μs, where the temperature slightly decreases to 129 °C. This phenomenon is probably attributed to a saturation effect or a redistribution of energy within the material, which impacts the heat transfer process. At the highest energy density (7.5 J·cm^−2^) and the longest pulse duration (25,000 μs), the temperature rises significantly to 148 °C. This substantial increase suggests that the combination of high energy density and extended pulse duration maximizes heat transfer to the perovskite film, resulting in a more pronounced temperature increase and, consequently, an impact on perovskite crystallization.

To confirm the formation of the perovskite structure, we analyzed the compositional and structural characteristics of the films using X-ray diffraction (XRD). Measurements were performed for perovskite films corresponding to the three conversion zones with the following annealing parameters: 1.01 J·cm^−2^/5000 μs, 3.52 J·cm^−2^/5000 μs, and 7.02 J·cm^−2^/500 μs. As shown in Figure 2i, the primary diffraction peaks were observed at 14.03°, 28.38°, and 31.75°, corresponding to the (110), (220), and (310) planes, respectively, confirming the formation of the MAPI structure [33]. Additionally, the presence of the (211) and (112) peaks in all films indicates a tetragonal crystal structure [34]. At lower energy densities (partially converted zone), the diffraction peak at 12.8° assigned to the (001) plane corresponds to residual $PbI_{2}$, suggesting the presence of small grains or incomplete crystallization. For the film treated with the optimum photonic annealing energy, maximum crystallization was achieved, as evidenced by more intense and sharper XRD peaks, reflecting larger crystallite size and improved crystalline orientation. However, beyond the optimum energy, the perovskite films degraded due to excessive energy input. This degradation is characterized by a decrease in peak intensity and the reappearance of the $PbI_{2}$ peak. Furthermore, Figure 2j compares the XRD spectra of TA and PC films. The results reveal that the intensity of the peaks is significantly higher for the films annealed using photonic curing at 3.52 J·cm^−2^/5000 μs compared to the reference film. This indicates that the photonic annealing process promotes enhanced crystallization. To gain a deeper insight into this crystallization phenomenon, we will analyze the influence of the photonic annealing parameters (energy density and pulse duration) on the granularity and microstructure of perovskite films in the following section.

**Figure 3:**
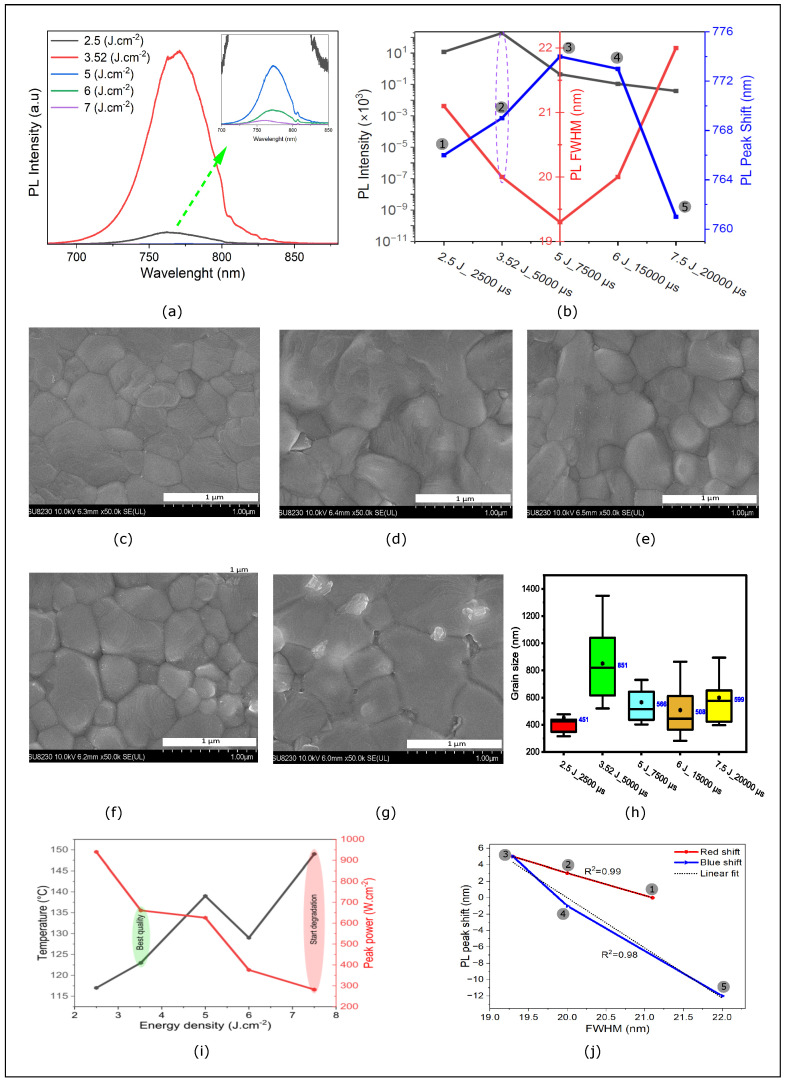
(**a**) PL spectra of photonically annealed perovskite films. (**b**) PL intensity, pic shift, and FHWM as a function of ED and pulse duration of photonically perovskite films. (**c**–**g**) SEM images of photonically annealed perovskite films at energy density and duration 2.5 J·cm^−2^/2500 μs, 3.52 J·cm^−2^/5000 μs, 5 J·cm^−2^/7500 μs, 6 J·cm^−2^/15,000 μs, and 7.5 J·cm^−2^/20,000 μs. (**h**) Grain size distribution vs. ED and pulse duration. (**i**) Peak power and simulated temperature as a function of annealing density energy for photonically annealed samples; PL peak shift as a function of peak width (FWHM) for photonically annealed sample. (**j**) The shift of the PL peak as a function of the FWHM of photonically annealed perovskite films at energy density and duration 2.5 J·cm^−2^/2500 μs, 3.52 J·cm^−2^/5000 μs, 5 J·cm^−2^/7500 μs, 6 J·cm^−2^/15,000 μs, and 7.5 J·cm^−2^/20,000 μs correspond to states 1, 2, 3, 4 and 5 respectively, states 1, 2 and 3 correspond to redshift (red line) and states 3, 4 and 5 correspond to blueshift (blue line), The dotted black line represents the fitted line.

PC of perovskite films involves using intense pulsed light to rapidly anneal the material. This process can enhance the crystallinity and reduce defects, leading to significant changes in PL spectra. Figure 3 depicts the results of PL and SEM measurements for CB-treated MAPI samples that were subjected to annealing at energy densities of 2.5, 3.52, 5, 6, and 7.5 J·cm^−2^, which correspond to the respective states 1, 2, 3, 4, and 5. As illustrated in Figure 3b, the PL peaks are found to be sensitive to both ED and pulse duration. The sample annealed at 3.5 J·cm^−2^ for 5000 μs exhibited the highest intensity peak with a relatively narrow FWHM. The higher PL intensity indicated a higher-quality material with a reduction in trap states and suppression of non-radiative recombination [35,36]. Additionally, a decrease in FWHM indicated a more uniform crystal size distribution and passivation of surface defects, reflecting increased crystallinity [37,38]. Another noteworthy observation is the shift in peak wavelength observed with the increase in ED. First, a redshift is observed at low energy density for annealed films from 2.5 J·cm^−2^ to 5 J·cm^−2^ (766 nm–774 nm). Beyond this ED, a transition to a blueshift occurs (774 nm–761 nm), which can be explained by the grain size distribution and phase transition in MAPI [39,40]. Figure 3c–g show SEM images of photonically annealed films at 2.5, 3.52, 5, 6, and 7.5 J·cm^−2^, respectively. These images illustrate the formation of uniform topographies with a large grain size distribution. As energy density increases, grain size reaches an average of 851 nm (Figure 3h), with a compact, uniform structure. Subsequently, grain size begins to decrease, and at higher energy densities, holes and cracks emerge (Figure 3g). Based on the simulation of temperature peaks at the surface of the perovskite film for each photonic treatment, the (7.5 J·cm^−2^/20,000 μs) treatment corresponds to a peak temperature of 148 °C. This high temperature causes film degradation, which can be explained by the easy release of methylammonium iodide (MAI) at this temperature [41,42]. This high temperature corresponds to the weak power peak of 282 W·cm^−2^, and the degradation is likely due to the long pulse duration of 20,000 μs (Figure 3i), which extends the annealing time and thus impacts the material stability or quality.

This observation aligns with the PL test presented above. This phenomenon can be attributed to the solvent evaporating too rapidly, leading to surface defects. Controlling the solvent evaporation rate is crucial for determining both the nucleation and growth rates of perovskite films [43,44,45]. When the solvent evaporation rate is high, the concentration of precursors increases rapidly. This leads to grain growth being restricted by the limited time and space available, resulting in a perovskite film that is fully coated but composed of uniformly small grains [44]. To achieve a perovskite film with high coverage, large grain size, and uniformity, it is essential to precisely control the solvent evaporation rate [46].

The shift in the PL peak as a function of its width (FWHM) provides an indication of the evolution of crystallinity when the energy is increased from the initial state (state 1) to the final state (state 5). Initially, an increase in energy density leads to a rapid red shift in the PL peak, accompanied by a simultaneous decrease in the FWHM. This behavior is attributed to the reduction in defects and the achievement of a significant degree of crystallization. During this phase, the evolutionary trend is perfectly linear, which can be explained by a decrease in the effect of phonon localization within the structure [47]. However, at higher energy densities, starting from stage 3, a blue shift in the PL peak is observed. Beyond this point, the appearance of new defects disrupts the crystalline structure, leading to a non-linear relationship between the PL peak shift and the FWHM width, indicating the onset of structural degradation (Figure 3j).

In other words, the photonic annealing coupled with CB antisolvent treatment created considerably larger and more uniform grain sizes. The UV-Vis absorption results, SEM topography, and PL measurements clearly demonstrate that the photonic annealing with the antisolvent used in this work can generate a PSC device with a power conversion efficiency (PCE) comparable to the standard TA method. As shown, films treated by photonic annealing have shown significant improvements in terms of crystallinity and defect density. In the following section, we will conduct a performance comparison study between a typical TA device and an optimized PC device (3.52 J·cm^−2^, 5000 μs) to evaluate the contribution of PC to the improvement of perovskite cell fabrication.

**Figure 4:**
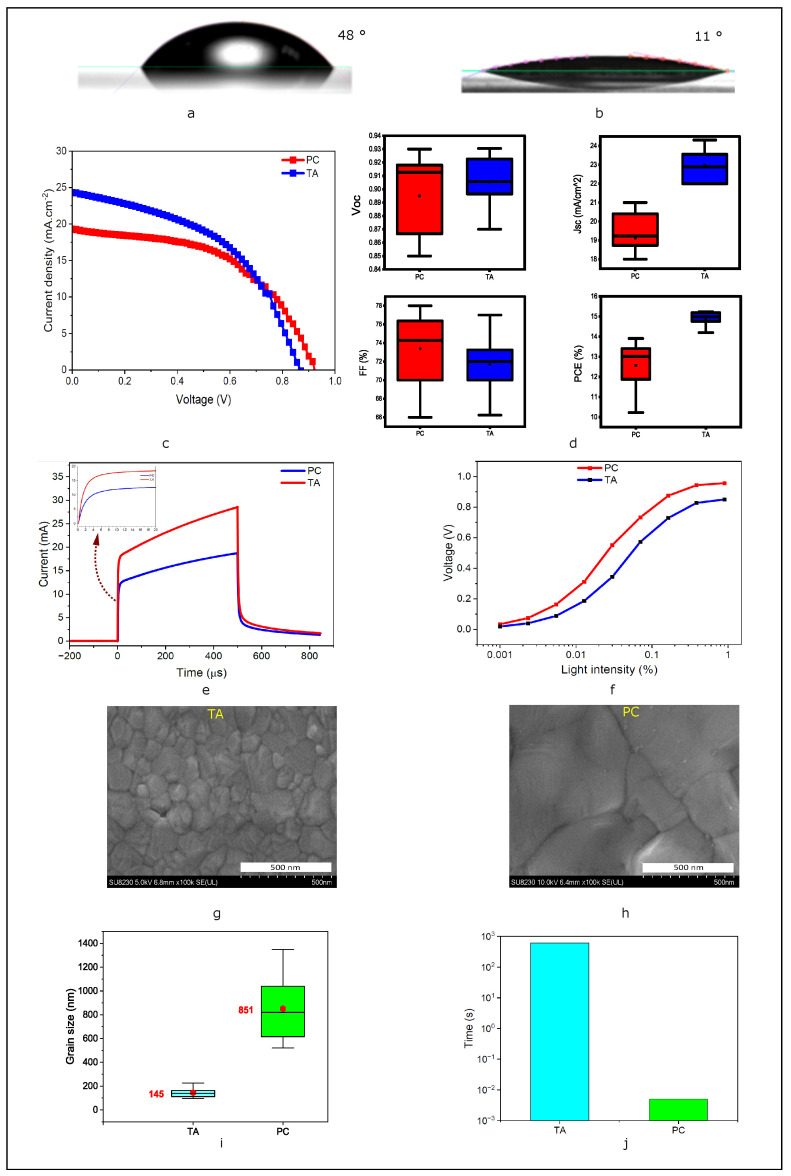
Contact angle measurement of the ink droplet on the $SnO_{2}$ coated substrate, (**a**) before plasma treatment and (**b**) after plasma treatment. (**c**) Device performance of champion of TA and PC, (**d**) Statistical distribution of the photovoltaic parameters for photonically and thermally annealed perovskite solar cells. (**e**) Transient photocurrent of the champion devices of TA and PC. (**f**) Open circuit voltage vs. light intensity of TA and PC champion devices. (**g**,**h**) SEM images of thermally and photonically annealed perovskite films. (**i**) Grain size distribution of thermally and photonically annealed perovskite films. (**j**) Comparison of the processing time for PC and TA.

Figure 4a,b shows contact angle measurements before and after $O_{2}$ plasma treatment of $SnO_{2}$ ETL layers. The decrease in contact angle signifies that this treatment has increased the surface energy and wettability of $SnO_{2}$, ensuring fewer surface defects, better adhesion, and greater uniformity of the perovskite layer [48]. This enhancement in surface properties leads to a more uniform and defect-free perovskite film, which is crucial for improving the overall efficiency and stability of the solar cell [49]. The plasma treatment, therefore, plays a significant role in optimizing the interface between the $SnO_{2}$ ETL, and the perovskite layer, contributing to enhanced device performance. Figure 4c,d present a performance comparison for typical TA and optimized PC devices (3.52 J·cm^−2^, 5000 μs). The data in Figure 4d show that open-circuit voltage (Voc) is similar and relatively high, indicating that the perovskite films are of better quality with reduced electron-hole recombination [50,51]. FF is slightly low for TA, probably due to its higher series resistance and the sensitivity of perovskite to moisture, light, and heat [52,53]. However, the variation in efficiency between the PC and TA samples is primarily attributed to the Jsc. We propose that this is due to interface effects arising from differences in topography, which could impact the transport of charge carriers [54,55]. Furthermore, all PC samples are processed in an ambient environment, which explains the relatively lower PCE of the PC samples compared to the TA samples due to their sensitivity to humidity. Figure 4e illustrates the transient photocurrent (TPC) comparison between the champion solar cells TA and PC. This comparison involves analyzing the time-dependent response of the photocurrent when the solar cells are exposed to light. The TA solar cell typically exhibits a fast rise time due to high-charge-carrier mobility and lower recombination rates. However, in the context of Figure 4f, which illustrates the effect of light intensity on the open-circuit voltage (Voc) for two types of solar cells (PC and TA), the greater sensitivity of Voc to light intensity in the PC solar cell compared to the TA solar cell suggests important differences in their photovoltaic behavior. This observation may be linked to the presence and impact of stronger trap states in TA than in PC [56]. Figure 4g,h show SEM images of thermally and photonically annealed perovskite films. Figure 4i illustrates that the average grain size in the PC samples is approximately six times larger than that in the TA samples. To our knowledge, no other grain size ratio has been reported to reach this magnitude of difference. This significant performance is mainly attributed to the photonic annealing process. Overall, the average PCE remains comparable to the TA process, even with the disadvantage of the environmental conditions. Figure 4j illustrates the significantly faster processing time of the photonic curing process, which uses 5000 μs, compared to the 10 min required for thermal annealing. This results in a 12,000-fold reduction in processing time, making perovskite cells produced by photonic curing compatible with R2R and high-throughput manufacturing.

## 4. Conclusions

Photonic annealing is an innovative technique applied to photoactive perovskite films to enhance their optoelectronic properties. This method uses light to induce structural and chemical changes in the film, promoting improved crystallinity and defect reduction. The results of the UV-Vis analysis enabled the mapping of the conversion zones of the perovskite films based on two independent parameters: energy density and pulse duration. PL measurements, coupled with SEM characterization, allowed us to define the optimal parameters for the conversion zone and to study the granularity and microstructure of photonically annealed films. A low radiant energy of 3.52 J·cm^−2^ and a pulse duration of 5000 μs provide the best performance. According to our treatment process, grain size increases with density up to a maximum, then begins to decrease with the presence of structural defects. The champion PCE of the PC (13.95%) is relatively modest compared to that of the TA (15.01%). However, the performance of the PSCs fabricated by TA is on average comparable to those fabricated by PC. The major advantage for PC is the reduction in energy and processing time by 120,000 times compared to TA. These improvements lead to increased photovoltaic performance, making photonic annealing a promising method for producing more efficient and durable perovskite-based solar cells.

## Data Availability

The data that support the findings of this study are available from the corresponding author upon reasonable request.

## Abbreviations

The following abbreviations are used in this manuscript:

ETL	Electron-transporting layer
PSC	Perovskite solar cell
TA	Thermal annealing
PC	Potonic curing
